# Supplementary figures and images for: Effect of mesoporous bioactive glass on odontogenic differentiation of human dental pulp stem cells
Source: PeerJ. 2021 Nov 23;9:e12421. doi: 10.7717/peerj.12421 (PMC8621711; doi:10.7717/peerj.12421)

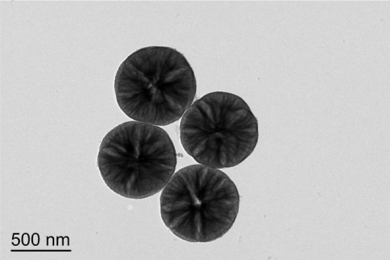

Supplement: Supplemental Information 1 [file peerj-09-12421-s001.zip › Raw data/Figure 1/Figure1 B.png]

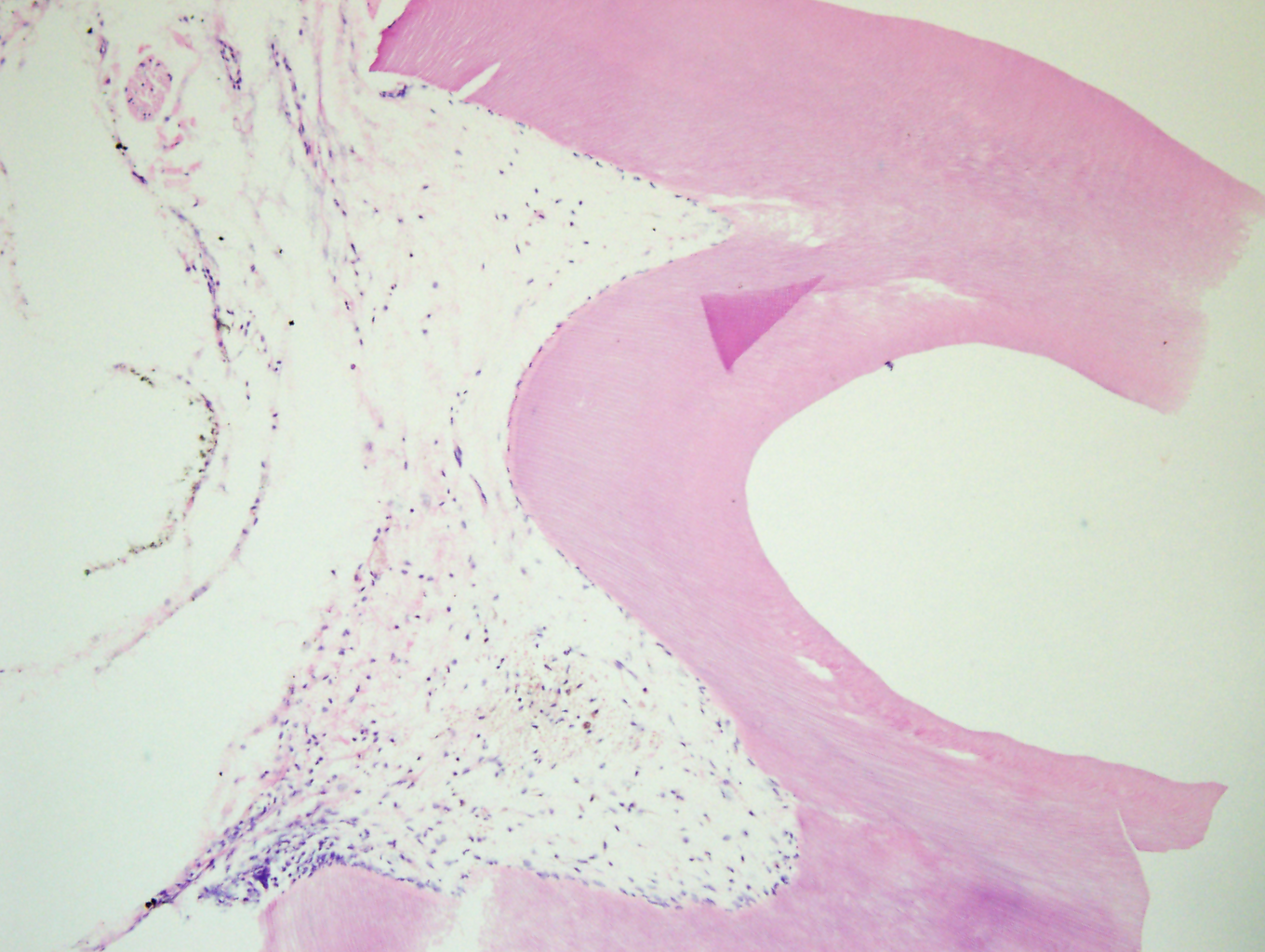

Supplement: Supplemental Information 1 [file peerj-09-12421-s001.zip › Raw data/Figure 3/Figure3-CTR/100X.tif]

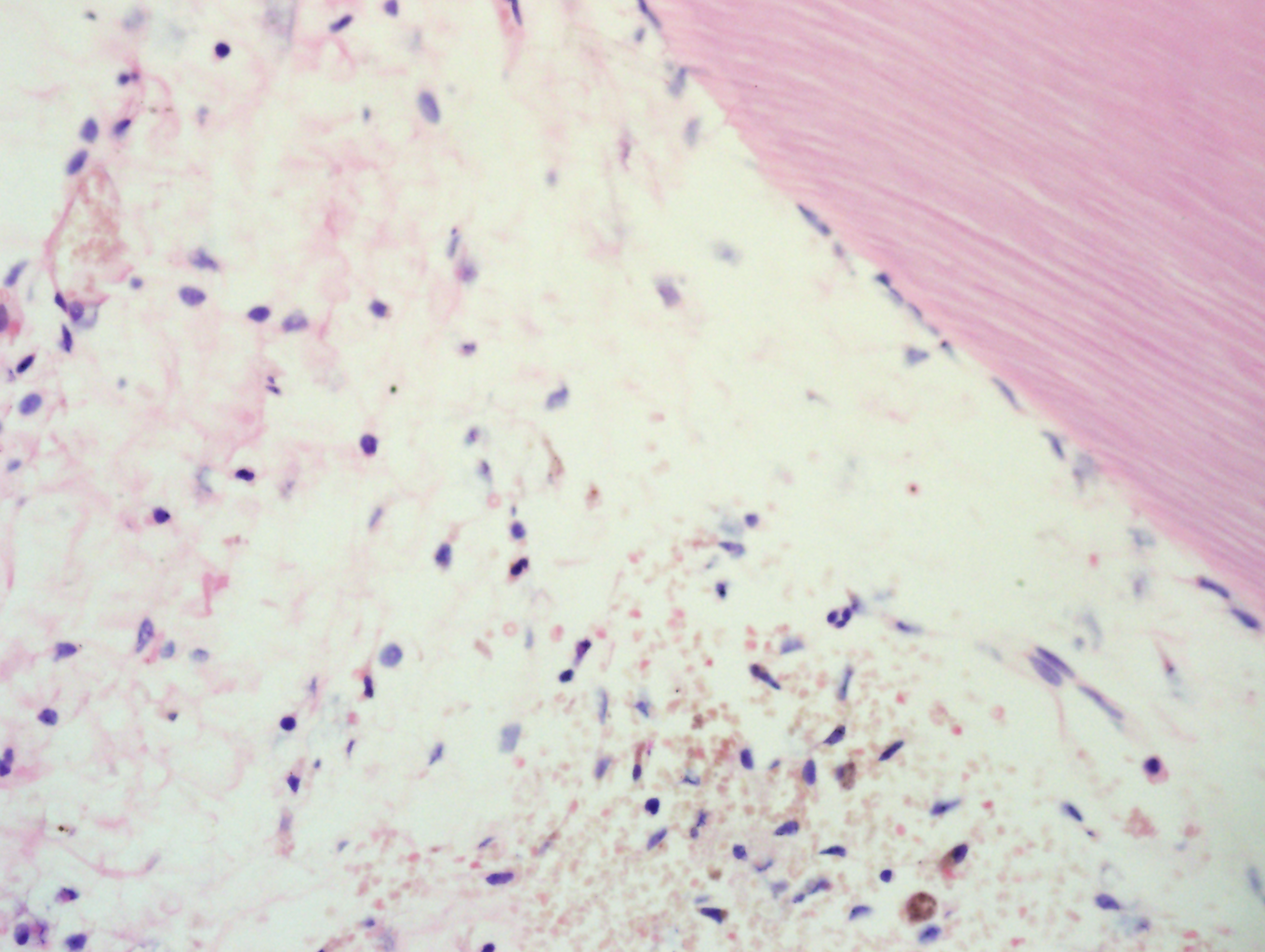

Supplement: Supplemental Information 1 [file peerj-09-12421-s001.zip › Raw data/Figure 3/Figure3-CTR/400X.tif]

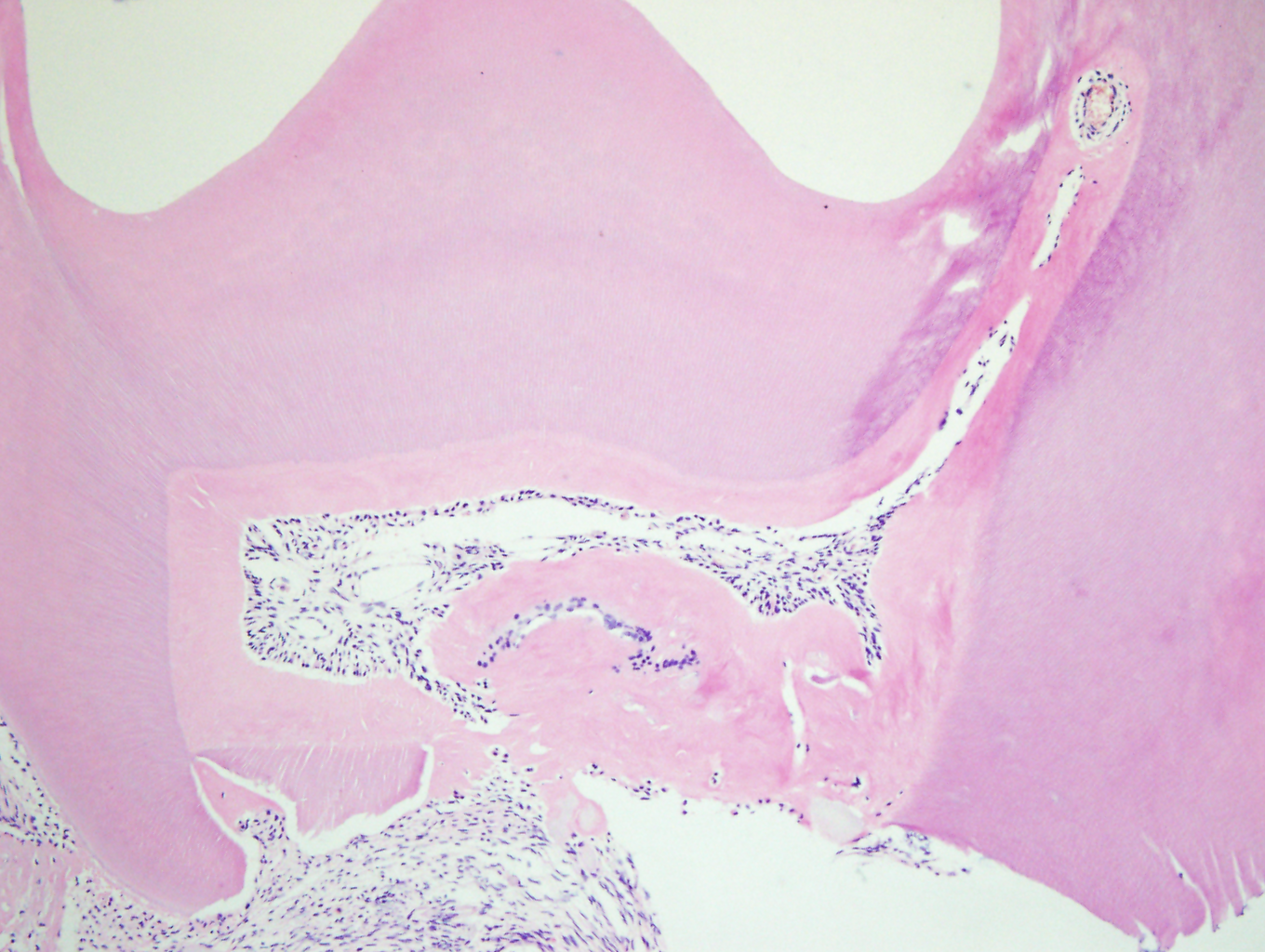

Supplement: Supplemental Information 1 [file peerj-09-12421-s001.zip › Raw data/Figure 3/Figure3-MBG/100x.tif]

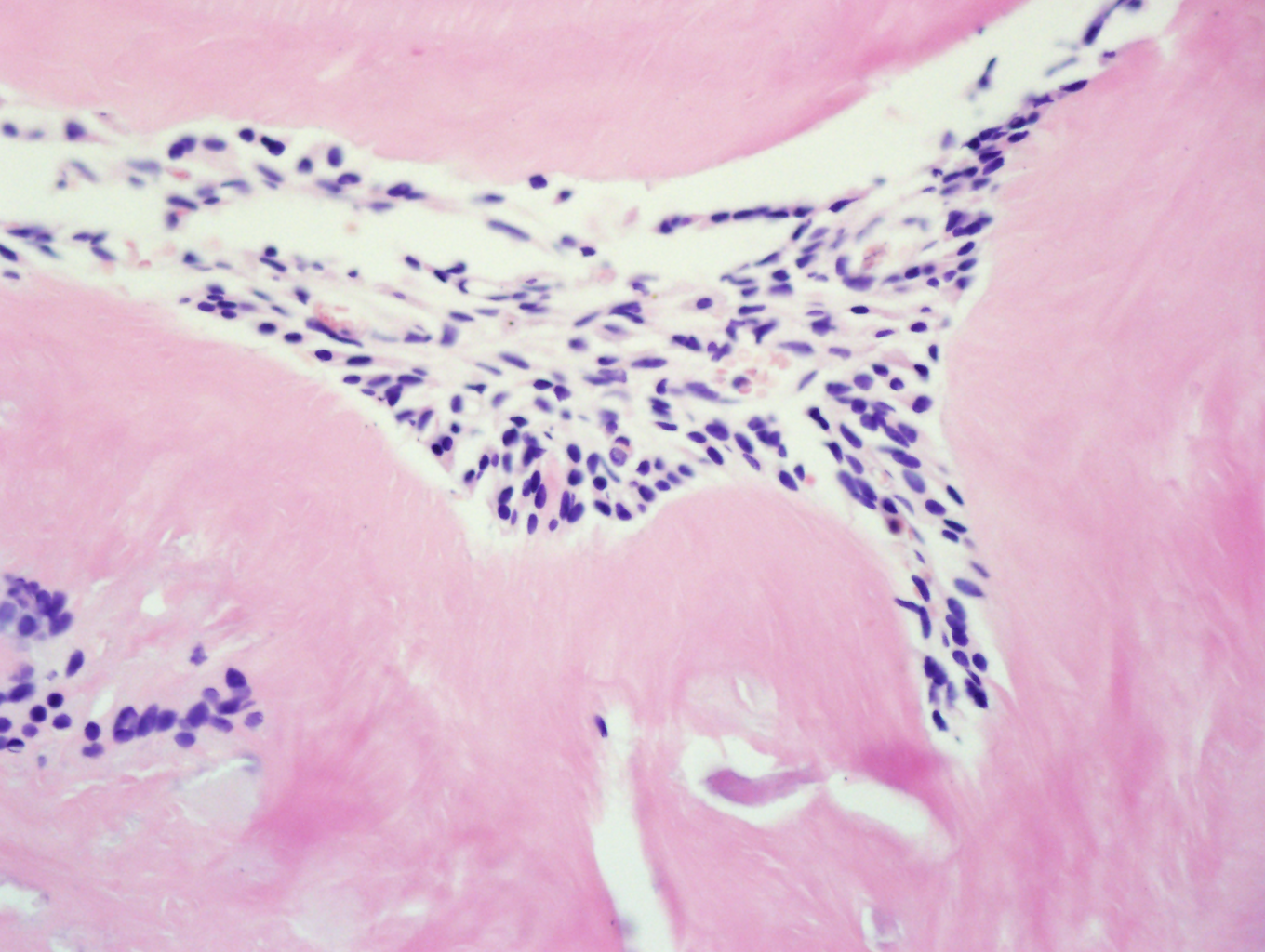

Supplement: Supplemental Information 1 [file peerj-09-12421-s001.zip › Raw data/Figure 3/Figure3-MBG/400x.tif]

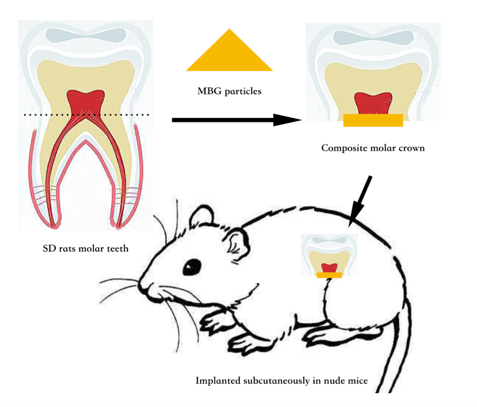

Supplement: Supplemental Information 2 [file peerj-09-12421-s002.png]
