# Supplementary material for: Effect of mesoporous bioactive glass on odontogenic differentiation of human dental pulp stem cells
Source: PeerJ. 2021 Nov 23;9:e12421. doi: 10.7717/peerj.12421 (PMC8621711; doi:10.7717/peerj.12421)
Supplement: Supplemental Information 1 [file peerj-09-12421-s001.zip › Raw data/Table2.doc]

**北京大学公卫学院中心仪器室测试报告**

| **送样单位** | | 北京大学口腔医院 | | | **送样日期** | 2017/9/4 | | |
| --- | --- | --- | --- | --- | --- | --- | --- | --- |
| **样品名称** | | 浸提液 | | | **送样人** | 朱林 | | |
| **测试方法** | | ICP-OES | | | **完成日期** | 2017/9/5 | | |
| **测试项目** | | Ca、P、Si含量 | | | | | | |
| **测试结果:** 样品名称 \| Ca(μg/mL） \| P(μg/mL） \| Si(μg/mL）; 1-BLK \| 65.39 \| 27.11 \| 0.076; 1-CTR \| 71.97 \| 27.23 \| 11.22; 1-60 \| 53.57 \| 19.65 \| 30.94 | | | | | | | | |
| **测试人员** | 刘雅琼 | | **审核人** | 欧阳荔 | | | **负责人** | 崔富强 |

1. 样品、样品名称及送样单位均由委托单位提供，本检测报告只对委托单位所送样品负责。
2. 未经本科室书面批准，任何单位或个人不得用本报告及本中心的名义作广告宣传。
